# Upper Extremity Function following Transradial Percutaneous Coronary Intervention: Results of the ARCUS Trial

**DOI:** 10.1155/2022/6858962

**Published:** 2022-09-06

**Authors:** Eva M. Zwaan, Elena S. Cheung, Alexander J. J. IJsselmuiden, Carlo A. J. Holtzer, Ton A. R. Schreuders, Marcel J. M Kofflard, J. Henk Coert

**Affiliations:** ^1^Department of Plastic Surgery and Reconstructive Surgery, University Medical Centre Utrecht, Utrecht, Netherlands; ^2^Departments of Heart and Lungs, University Medical Centre Utrecht, Utrecht, Netherlands; ^3^Department of Cardiology, Amphia Hospital, Breda, Netherlands; ^4^Department of Plastic Surgery and Hand Surgery, Albert Schweitzer Hospital, Dordrecht, Netherlands; ^5^Department of Plastic Surgery and Reconstructive Surgery, Erasmus Medical Centre, Rotterdam, Netherlands; ^6^Department of Cardiology, Albert Schweitzer Hospital, Dordrecht, Netherlands

## Abstract

**Objectives:**

To determine the incidence of upper extremity dysfunction (UED), after a transradial percutaneous coronary intervention (TR-PCI).

**Background:**

Transradial approach (TRA) is the preferred approach for coronary interventions. However, upper extremity complications may be underreported.

**Methods:**

The ARCUS was designed as a prospective cohort study, including 502 consecutive patients admitted for PCI. Patients treated with transfemoral PCI (TF-PCI) acted as a control group. A composite score of physical examinations and questionnaires was used for determining UED. Clinical outcomes were monitored during six months of follow-up, with its primary endpoint at two weeks.

**Results:**

A total of 440 TR-PCI and 62 control patients were included. Complete case analysis (*n* = 330) at 2 weeks of follow-up showed that UED in the TR-PCI group was significantly higher than that in the TF-PCI group: 32.7% versus 13.9%, respectively (*p*=0.04). The three impaired variables most contributing to UED were impaired elbow extension, wrist flexion, and extension. Multivariate logistic regression showed that smokers were almost three times more likely to develop UED.

**Conclusions:**

This study demonstrates that UED seems to occur two times more in TR-PCI than in TF-PCI at 2 weeks of follow-up. However, no significant long-term difference or difference between the intervention arm and the contralateral arm was found at all timepoints.

## 1. Introduction

The transradial artery approach (TRA) is the preferred approach for percutaneous coronary interventions (PCI) with reported safer postprocedural haemostasis of the arterial access, shorter hospital admissions, earlier ambulation, and better patient comfort [1–3]. Furthermore, a crucial advantage of the TRA is reduced mortality across the entire range of coronary artery disease [4]. However, complications resulting in upper extremity dysfunction (UED) may be underreported due to late onset of symptoms, lack of recognition during follow-up, or reluctance of operators to acknowledge and report complications [5, 6].

Upper extremity disorders have various social and economic consequences that are far-reaching and have a profound effect on matters central to patients' lives [7–9].

The impairment of an upper extremity following TRA could be of great consequence for managing activities of daily livings (ADLs) performance and vocational health and should therefore be concerned in the decision making for percutaneous treatment approach.

Current knowledge is insufficient in objectifying the occurrence of UED and access-site complications following transradial percutaneous coronary intervention (TR-PCI). This study evaluates the effect of TR-PCI on upper extremity function using endpoints designed by hand specialists.

## 2. Methods

### 2.1. Study Design

The aim of the ARCUS (Effects of trAnsRadial perCUtaneouS Coronary Intervention on Upper Extremity Function) was to determine the effects of TR-PCI on upper extremity function, the occurrence of access-site complications, and the safety of TR-PCIs. Subjects were included if they had a palpable radial artery and if Doppler ultrasound examination confirmed nonocclusive flow. Exclusion criteria were (1) radial artery occlusion objectified by Doppler ultrasound and (2) incapability to accomplish measurements due to comorbidities [10]. After a protocol amendment (inclusion *n* = 238), a transfemoral approach (TFA) control group was included. The protocol and amendment were approved by both the regional ethics committee (Toetsingscommissie Wetenschappelijk Onderzoek Rotterdam (TWOR), Rotterdam, Netherlands) and the local ethics committees of the participating hospitals.

### 2.2. Patient Population

Patients were enrolled between January 2014 and July 2018 in two high-volume centres in the Netherlands (Table 1). The target inclusion was 500 patients: 400 TRA patients and 100 TFA patients.

### 2.3. Upper Extremity Assessments

Written informed consent was obtained. Trained research nurses performed the examinations specified in a previously published study protocol [10] bilaterally. Strength of the key grip, palmar grip, flexion, and extension of the wrist and elbow were measured. Strength measurements were conducted three times and results were averaged. If a subject was unable to perform three strength measurements because of pain or other symptoms, an average of the obtained measurements was included. Sensibility of the digits according to the Weinstein Enhanced Sensory Test (WEST), circumference of the hand and forearm, and Doppler velocimetry of the radial and ulnar artery were measured. All measurements were performed according to the recommendations of the American Society of Hand Therapists (ASHT) [11] and were taken before the procedure and 24 hours, two weeks, one month, and six months after the procedure. Functional status was assessed using self-administered questionnaires regarding pain, using the Numeric Rating Scale for Pain (NRSP) [12], disabilities of the upper extremity, using the Disabilities of the Arm, Shoulder, and Hand (DASH) questionnaire [13], and Carpal Tunnel Syndrome, using the Boston Carpal Tunnel Questionnaire (BCTQ) [14].

Interim analysis revealed that the questionnaires, especially the DASH questionnaire, and the rigorous cut-off points of the strength measurements yielded false positive results for both extremities [15]. Thus, the questionnaires were excluded and the cut-off values for those measurements were modified to percentage instead of absolute change (Table 1) [15]. The modified primary endpoint was applied for the final analysis.

Percutaneous coronary intervention operators adhered to the international practice guidelines for the performance of PCI [16]. Following local anaesthesia, a 6F introducer sheath (Terumo) was inserted in the radial artery using the (modified) Seldinger technique. A saline solution with 5 mg verapamil, 200 *µ*g nitroglycerine, and 100 IU/kg heparin was administered. All procedures were performed with a 6F hydrophilic guiding catheter (PRIMUM, Wellinq, Leek, Netherlands). This hydrophilic coating provides more accurate and precise tip positioning in ostial lesions and a 1 : 1 torque control of the tip. The outer diameter is downsized compared to a 7 Fr catheter, while the large inner lumen offers expanded device compatibility with larger profile devices. Furthermore, it has a small atraumatic soft tip to minimize vessel damage [17].

Fractional flow reserve measurements, balloon angioplasty, or stent implantation was performed in ≥1 coronary artery. After the procedure, haemostasis was obtained using compression device (Terumo, Medical Corporation, Tokyo, Japan) with 13 cc air. After four hours, the device was gradually deflated completely in two hours, after which 4 cc of air was reinjected, leaving the device applied for 24 hours after the procedure.

Data collection for this study was performed using OpenClinica software, version 3.14 (OpenClinica LLC and collaborators, Waltham, MA, USA).

### 2.4. Primary Endpoint

The primary endpoint was presence of UED in the intervention extremity at two weeks of follow-up. In absence of a comprehensive definition of UED, hand experts formulated for this study a definition of UED composed of eight criteria (Table 1). Presence of UED was defined as ≥ 2 criteria at follow-up. A study protocol was published earlier with elaborate substantiation for this endpoint [10].

### 2.5. Secondary Endpoints

Secondary endpoints were UED at one and six months of follow-up, changes in strength and sensibility, referrals to a hand centre, and Major Adverse Cardiovascular Events (MACE), access-site complications, such as active bleedings and minor (<5 cm in diameter) or major (>5 cm in diameter) hematomas, and radial artery occlusion (RAO) [10].

### 2.6. Statistical Analyses

The results are presented as means ± SD, medians ± IQR, counts, and percentages. Measurements of the TRA group were categorized in intervention extremity and the contralateral, nonintervention extremity and measurements of the TFA group were categorized in right and left upper extremity. Between-group differences of dichotomous variables and differences in the two upper extremity scores (intervention extremity vs. nonintervention extremity or right vs. left) were analysed using Chi-squared test.

To handle incomplete data, two types of missing data analyses were conducted, complete-case analysis (i.e., all measurements composing UED were complete) and available-case analysis (i.e., enough measurements were present to qualify for UED (≥2 criteria present)) (Table 2). Complete-case analysis gives unbiased results if patients with and without missing data are similar in characteristics, but also results in loss of precision of reported estimates and reduced statistical power. Available-case analysis uses more available information but will also reduce precision of estimates.

The independent sample *t*-test was used for normally distributed variables, and the Mann–Whitney *U* test was applied for nonnormally distributed variables.

Furthermore, the Friedman's test was used to compare longitudinal continuous measurements and the Cochran's *Q* test was used to compare longitudinal categorical measurements across all timepoints. Wilcoxon signed-rank test and Holm–Bonferroni correction were used to compare repeated strength measurements at each executive follow-up versus baseline.

To assess for the potential confounding effects of baseline characteristics on missing data, a stratified propensity score was used to create comparable cohorts of patients. The propensity score for comparing the approach groups (radial or femoral) was divided in quintiles, generating a composite test statistic which was analysed using the Mantel–Haenszel test on the stratified contingency table.

To predict UED, baseline and procedure specific variables were evaluated in a univariate analysis, where variables with *p*-value <0.30 were identified as potential predictors.

Multivariate logistic regression analysis, using the “Enter” method, was performed to identify variables that independently could predict UED at two weeks following TR-PCI.

Linear regression analysis was performed to test for a correlation between the type of approach and severity of UED.

Statistical analyses were performed with SPSS for Mac version 26, R for Mac version 3.6.3, and MATLAB (version R2013b; the MathWorks, Natick, MA, USA).

All statistical tests used a two-sided statistical significance level of 5%.

## 3. Results

**Figure 1:**
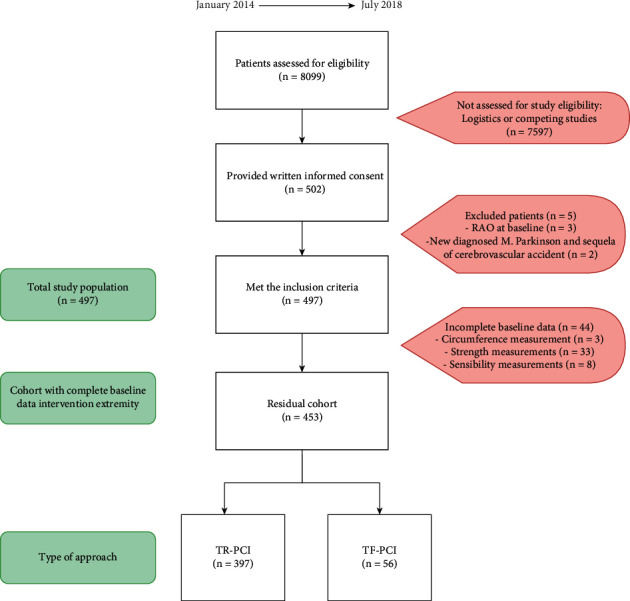
Flowchart study enrolment. RAO: radial artery occlusion, TR-PCI: transradial percutaneous coronary intervention, and TF-PCI: transfemoral percutaneous coronary intervention.

A total of 502 patients were enrolled: 440 TRA patients and 62 TFA patients (Figure 1). The right radial artery was used in 98.5% of cases. The protocol was violated in three TFA patients because of RAO at baseline and in two TRA patients because of preexisting impairing disease (Figure 1).

Complete baseline data was missing in 44 (8.9%) patients, whereupon the primary endpoint could not be calculated in these patients (Figure 1). Data was incomplete for all follow-up visits in 26 patients (5.2%), due to withdrawal or lost to follow-up. Complete baseline and follow-up data were available in 294 for the intervention extremity only, whereas complete follow-up data of both extremities was collected in 163 enrolled patients.

### 3.1. Baseline Characteristics

Baseline characteristics are presented in Table 3. There were no statistically significant differences between the two approach groups.

### 3.2. Primary Endpoint

Complete case analysis, analysing patients with complete data for measurements composing UED, showed that the occurrence of UED in the TRA group did only significantly differ from the TFA group at two weeks after procedure (Table 2). Ninety-six patients (32.7%) in the TRA group and five patients (13.9%) in the TFA group had UED in the intervention extremity (*p*=0.034). This evolved in 134 patients (44.4%) and 24 (57.1%) at six-month follow-up, respectively (Table 2).

When specifying the three most significant contributing factors for UED at two weeks in the TRA group, elbow extension (*n* = 41 (42.7%)), wrist flexion (*n* = 47 (48.9%)), and wrist extension (*n* = 39 (40.6%)) were found to be the most common. In the TFA group, the main contributing variables also were elbow extension, wrist flexion, and wrist extension, respectively, in 60%, 80%, and 60% of patients.

McNemar's test showed no difference in UED between the intervention/right extremity and the contralateral extremity for all follow-up moments (*p* > 0.05).

To adjust for possible measured confounders between approach groups for missing data, a stratified propensity score was made. After assigning each individual with a propensity score, the TRA and TFA group was well balanced in terms of all observed covariates across strata. No measured confounding was responsible for the loss to follow-up at six months, as determined by stratified propensity score analysis (*p* > 0.05).

### 3.3. Follow-Up

Cochran's *Q*-test indicated a significant increase (+12.4%) of UED in the intervention extremity of the TRA group during follow-up ( *χ*^2^(2) = 8.8, *p*=0.01). In the contralateral extremity, there was also a significant increase (+14.3%) ( *χ*^2^(2) = 8.6, *p*=0.01).

Cochran's *Q*-test indicated that there was a significant increase in UED in the right upper extremity of the TFA group during follow-up (*χ*^2^(2) = 18.8, *p* < 0.001).

### 3.4. Distinct Measurements

**Figure 2:**
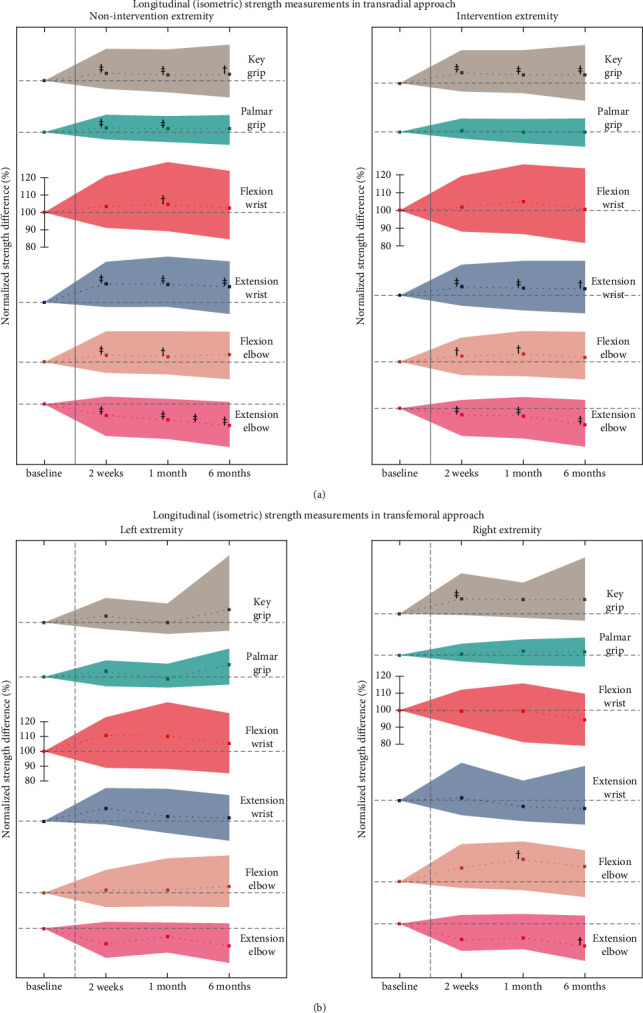
Longitudinal strength measurements. Longitudinal evolution of strength measurements for both extremities in the transradial ((a) *n* = 404) and transfemoral ((b) *n* = 53) groups. Values are median ± IQR. Values are normalized per patient by each baseline measurement. The horizontal gray dotted line indicates “no change.” The single *y*-scale holds for all graphs per subplot. Change with respect to baseline is indicated at the posttreatment timepoints, and with respect to the previous time point in between the posttreatment timepoints, measured with Wilcoxon signed-rank test and Holm–Bonferroni correction. ^‡^*p* < 0.01 and ^†^*p* < 0.05.

Individual (strength) measurements were assessed (Figure 2) via a Friedman's test and showed predominantly an increase in strength during follow-up. However, Friedman's test for repeated measures showed an increase for the circumference of the intervention forearm (*χ*^2^(3, *n* = 270) = 21.0, *p* < 0.001), and a similar increase was demonstrated in the contralateral arm (*χ*^2^(3, *n* = 259) = 9.9, *p* < 0.019). Furthermore, a decrease in elbow extension strength was found across all timepoints (*χ*^2^(3, *n* = 256) = 40.4, *p* < 0.001) (Figure 2).

This decrease in strength was clinically relevant, with an average drop of 75 newtons at two weeks and 133 newtons at six months.

The nonintervention extremity also showed a decrease of the elbow extension strength (*p* < 0.001), with a steeper drop of 101 newtons on average after two weeks and 160 newtons after six months (Figure 2).

### 3.5. Access-Site Complications

Access-site complications were not different between approaches, except for more minor hematoma at day-one postprocedural in the TRA group (*p*=0.01) (Table 4). Hematomas were not different between types of punctures ((modified) Seldinger's technique), number of punctures until successful access, and number of needle movements. There was, however, a difference between operators (*p*=0.046). Sixteen (4.9%) TRA patients had RAO during follow-up, of which over time 14 (88%) recanalized (Table 4). In the TRA group, 13 patients (3.2%) were referred to the hand specialist two weeks after procedure, whereas no patients in the TFA group were referred.

### 3.6. MACE

Eight patients had a myocardial infarction and seven patients required revascularisation. There was no difference in MACE between approaches (*p*=0.94).

### 3.7. Regression Analysis

Univariate analysis showed that female gender (*p*=0.005), lower height (*p*=0.005), smoking (*p*=0.008), and family history for cardiovascular disease (*p*=0.04) were associated with UED. Other potential UED predictors (*p* < 0.30) were lower weight, hypertension, and operator.

Multivariate logistic regression identified factors that independently could predict UED two weeks after TR-PCI. The model with seven variables was significant (*χ*^2^(17, *n* = 325) = 38.912, *p*=0.002), indicating that the model was able to distinguish between respondents with and without UED. Only three of the independent variables made a significant contribution to the model: smoking (OR 2.73, 95% CI 1.41–5.28), hypertension (OR 1.9, 95% CI 1.04–3.09), and positive family history for heart disease (OR 1.74, 95% CI 1.04–2.91). The strongest predictor of UED was smoking, recording an odds ratio of 2.7. Smokers were almost three times more likely to demonstrate UED than nonsmokers.

The one-way ANOVA regression model failed to reject the null hypothesis of equal variances; however, it showed strong evidence for the effect of approach type on the variance in severity of UED at two weeks (*F* (1, 327) = 3.840, *p* = 0.051). In this case, only 1% of the total variation in severity of UED could be explained by the type of approach (*R*^2^ = 0.012).

## 4. Discussion

The ARCUS study provides insight in access-site complications and explores and tries to characterize the level of impairment associated with TR-PCI.

This study demonstrated the presence of UED in 32.7% of patients undergoing TR-PCI after two weeks and in 44.4% of patients after six months of follow-up. At two weeks, this was statistically significant different from the TFA group. Remarkably, in both groups, UED occurs bilateral and increases over time. No significant long-term difference could be demonstrated, most likely due to reduced power caused by missing data. This is partially in consensus with a previous study, which described upper limb function assessed using self-reported questionnaires at 1 month. Upper limb function after the transradial catheterization was not different from patients with the transfemoral catheterization [18]. However, there were no objective measurements, there was no data available for UED at 2 weeks or 6 months, and the procedure did not entail (bulky) interventions.

The decline in the stand-alone measurement results was clinically relevant [10]; however, we hypothesize that our primary endpoint is overly stringent in classifying UED, and only two impaired criteria may not induce a debilitating level of impairment.

A clinically important reduction of extension strength of the elbow in both extremities was observed after two weeks and six months. Extension of the elbow is powered by the triceps brachii muscle, which is essential for weight bearing, mobilization (getting out of the chair), and is innervated by the radial nerve [19]. Long-term inactivity, a period of ≥4 weeks of insufficient training, appears to lead to 17% decrease strength. However, maximal strength on the other hand was preserved [20].

In this specific patient group, short-term UED is a consequence of the intervention and long-term UED could be partially caused by temporarily cessation of physical activity shortly after the myocardial infarction awaiting cardiac rehabilitation. This could contribute to a general deterioration in muscle condition as both the contralateral arm and the TFA group demonstrated dysfunction.

In the intervention, extremity circumference of the forearm was significantly increased over time. However, a similar increase was measured in the contralateral extremity, insinuating an (additional) systematic cause, a possible side effect of calcium channel blockers or heart failure [21].

There was no clinically or statistically significant difference in muscle force of the hand following TR-PCI measured by the palmar and pinch grip. This is in consensus with the findings of the HANGAR study of Sciahbasi et al., who found no reduction in hand and finger strength at 30 days of following a transradial percutaneous coronary procedure. Furthermore, radial artery occlusion after TR-PCI was not associated with a reduction in strength [22].

A possible explanation for UED at two weeks could be intervention induced trauma of the hand and wrist (e.g., sheath-to-artery mismatch and prolonged application of a radial artery pressure device). Female gender and lower body length were more associated with UED, which could be explained by women generally having a smaller radial artery diameter than men, resulting in more sheath-to-artery mismatch [23].

Smokers were almost three times more likely to develop UED than nonsmokers. Smoking influences age-related loss of muscle mass and strength, is associated with a higher risk of musculoskeletal pain, and impairs the muscle metabolism by increasing inflammation and oxidative stress, inducing skeletal muscle damage [24–26]. Other risk factors hypertension and family history of cardiovascular disease. Our suggestion is to consider the multivariate variables for risk stratification of complications following TR-PCI.

### 4.1. Limitations of Our Study

In 44 cases, baseline data were missing regarding circumference of the extremity and strength measurements. Not all data could be collected due to the inconvenient drip placement. Analysis of missing data during follow-up showed no selective loss to follow-up, indicating that our results were not biased by measured confounding. Additionally, the authors are aware that during this ongoing study, in 2018 Aminian et al. [27] and in 2019 Bernat et al. [28] gained insights regarding haemostasis strategies and radial artery occlusions. These strategies were not applied in order to keep the study group homogenous.

Future research regarding TR-PCI should focus on risk stratification for UED. Refining techniques through downsizing coronary interventional material and equipment or sheetless catheters remains an important goal. Finally, attention should be paid to improve haemostasis methods and balancing the risk of approach while taking the patient's dexterity and anatomy into consideration.

## 5. Conclusion

In this study, the presence of UED seems to occur two times more in radial access (TR-PCI) than in femoral access (TF-PCI) at two weeks of follow-up. The three impaired variables contributing the most for UED were impaired elbow extension, wrist flexion, and extension. However, no significant long-term difference was found, most likely caused by reduced power due to missing data. There was no difference between intervention and contralateral arm at all points in time. We did see a clinically relevant decrease in triceps function in both groups at six months, which could be caused by cessation of physical activities. We advise awareness for, especially early, upper extremity complications and suggest referring to a hand specialist when they occur. Prevention using slender PCI and optimal patent haemostasis of the radial artery could also reduce UED.

## Figures and Tables

**Table 1:** Composed primary endpoint: Upper Extremity dysfunction at two weeks.

Criteria	Initial primary endpoint^*∗*^	Current primary endpoint^∗∗^
≥1 point increase in the symptom-severity or the functional-status score of the BCTQ	✔	✖
≥15% increase in the DASH compared to baseline	✔	✖
Increased NRSP score regarding the upper extremity of ≥2 points compared to baseline	✔	✔
Absent signal of the radial artery during Doppler ultrasound examination.	✔	✔
Strength compared to baseline		
≥ 60 N decrease in palmar grip strength	✔	≥15% decrease
≥ 12 N decrease in key grip strength	✔	≥15% decrease
≥ 15% decrease in flexion and extension strength of the elbow and wrist	✔	✔
≥1 filament increase in sensibility of the hand according to the WEST, compared to baseline	✔	≥2 filaments increase
≥1 cm increase of circumference of the hand, compared to baseline.	✔	≥2 cm increase compared to baseline
≥1 cm increase of circumference of the forearm, compared to baseline	✔	≥2 cm increase compared to baseline

^∗∗^Dysfunction present: ≥1 point increase in BCTQ or ≥2 criteria, ^∗∗^Dysfunction present: ≥2 criteria, BCTQ: Boston Carpal Tunnel Questionnaire, DASH: Disabilities of Arm, Shoulder and Hand, NRSP: Numeric rating scale for pain, and WEST: Weinstein Enhanced Sensory Test.

**Table 2:** Primary endpoint and long-term upper extremity dysfunction during follow-up in different approaches.

			2 weeks (*n* = 294)	1 month (*n* = 309)	6 months (*n* = 302)	Cochrane *Q*-test	*P*-value trend
TRA	Intervention extremity	Complete case analysis	96 (32.7%)	99 (32.0%)	134 (44.4%)	11	0.004
		Partial case analysis	115 (36.7%)	121 (36.6%)	159 (48.6%)	8.8	<0.012

			2-week primary endpoint (*n* = 36)	1 month (*n* = 44)	6 months (*n* = 42)	Cochrane *Q*-Test	*P*-value trend
TFA	Right upper extremity	Complete case analysis	5 (13.9)	14 (31.8%)	24 (57.1%)	22.7	<0.001
		Partial case analysis	8 (20.5%)	17 (36.2%)	26 (59.1%)	18.8	<0.001

*χ* ^2^	*P*-value^*∗*^	Complete case analysis	0.034	1.00	0.164		
		Partial case analysis	0.068	1.00	0.253		

Analysis per follow-up. Values are *n* (%). Chi-square test (*χ*^2^) with Yates Continuity Correction was used. ^*∗*^ Indicates statistically significant difference between intervention extremity in TRA group and right extremity in TFA group. TFA: transfemoral approach, TRA: transradial approach.

**Table 3:** Baseline characteristics of the total study sample.

		All patients *N* = 453	TRA *N* = 397	TFA *N* = 56	*P*-value
Men		355 (78.4%)	309 (77.8%)	46 (82.1%)	0.576
Age, y		65.5 ± 10.1	65.3 ± 10.1	66.6 ± 10.6	0.356
Body mass index		27.0 ± 5.0	27.1 ± 5.0	26.4 ± 5.8	0.239
Height		175.6 ± 9.1	175.6 ± 9.1	174.9 ± 9.3	0.589
Smoking	Current	76 (16.8%)	67 (16.9%)	9 (16.1%)	0.603
	Previous	232 (51.2%)	206 (51.9%)	26 (46.4%	
	Never	143 (31.6%)	122 (30.7%)	21 (37.5%)	

Hypertension		244 (53.9%)	220 (55.4%)	24 (42.9%)	0.097
Dyslipidaemia		190 (41.9%)	159 (40.1%)	31 (55.4%)	0.046
Diabetes mellitus		100 (22.1%)	85 (21.4%)	15 (26.8%)	0.474
Family history of heart disease		225 (49.7%)	191 (48.1%)	34 (60.7%)	0.087
Preexistent hand disease in intervention arm		247 (54.5%)	216 (54.4%)	31 (55.4%)	1.00
Previous TR-PCI		133 (29.4%)	119 (30.0%)	14 (25.0%)	0.543
Right hand dominance		403 (89.0%)	351 (88.4%)	52 (92.9%)	0.499
Prescribed calcium antagonists		323 (71.3%)	288 (72.5%)	35 (62.5%)	0.933

Values are mean ± SD, median ± IQR or *n* (%). *P* value for difference between sites. Chi-squared test for trend, using linear by linear to calculate *p*-value for three categories, TFA: transfemoral approach, TRA: transradial approach, and TR-PCI: transradial percutaneous coronary intervention.

**Table 4:** Access-site complications following percutaneous coronary intervention.

	TRA *N* = 401				TFA *N* = 56				*P*-value
	Day 1	2 weeks	1 month	6 months	Day 1	2 weeks	1 month	6 months	
Minor bleeding	23 (5.7%)	0 (0.0%)	0 (0.0%)	0 (0.0%)	1 (1.8%)	0 (0.0%)	0 (0.0%)	0 (0.0%)	NS
RAS	6 (1.5%)	23 (5.7%)	10 (2.5%)	14 (3.5%)	1 (1.8%)	2 (3.6%)	3 (5.4%)	1 (1.8%)	NS
RAO	3 (0.7%)	6 (1.5%)	4 (1.0%)	3 (0.7%)	1 (1.8%)	0 (0.0%)	1 (1.8%)	1 (1.8%)	NS
Minor hematoma	66 (16.5%)	58 (14.5%)	11 (2.7%)	0 (0.0%)	0 (0.0%)	6 (10.7%)	0 (0.0%)	0 (0.0%)	NS
Major hematoma	11 (2.7%)	36 (9.0%)	3 (0.7%)	0 (0.0%)	1 (1.8%)	4 (7.1%)	0 (0.0%)	0 (0.0%)	NS
Swelling	17 (4.2%)	5 (1.2%)	0 (0.0%)	1 (0.2%)	0 (0.0%)	0 (0.0%)	0 (0.0%)	0 (0.0%)	NS
Blister	1 (0.2%)	1 (0.2%)	0 (0.0%)	0 (0.0%)	0 (0.0%)	0 (0.0%)	0 (0.0%)	0 (0.0%)	NS
Wound infection	0 (0.0%)	1 (0.2%)	2 (0.5%)	0 (0.0%)	0 (0.0%)	0 (0.0%)	0 (0.0%)	0 (0.0%)	NS
Hand-specialist referral	0 (0.0%)	13 (3.2%)	17 (4.2%)	24 (6.0%)	0 (0.0%)	0 (0.0%)	1 (1.8%)	3 (5.4%)	NS

*P* value for difference between sites. Chi-squared test for trend, using linear by linear to calculate *p*-value for three categories. RAO: radial artery occlusion, RAS: radial artery stenosis, TFA: transfemoral approach, and TRA: transradial approach.

## Data Availability

Data management for this study was performed using OpenClinica software, version 3.14 (OpenClinica LLC and Collaborators, Waltham, MA, USA).

## Abbreviations:

ADL: Activities of daily living
ASHT: American Society of Hand Therapists
ARCUS: Effects of trAnsRadial perCUtaneouS Coronary Intervention on Upper Extremity Function
BCTQ: Boston Carpal Tunnel Questionnaire
DASH: Disabilities of the Arm, Shoulder and Hand
MACE: Major adverse cardiovascular events
NRSP: Numeric rating scale for pain
PCI: Percutaneous coronary intervention
RAO: Radial artery occlusion
TFA: Transfemoral approach
TRA: Transradial approach
TR-PCI: Transradial percutaneous coronary intervention
TWOR: Toetsingscommissie Wetenschappelijk Onderzoek Rotterdam
UED: Upper extremity dysfunction
WEST: Weinstein enhanced sensory test.
